# 1,4-Bis[(3,5-dimethoxy­phen­yl)ethyn­yl]benzene

**DOI:** 10.1107/S160053680900155X

**Published:** 2009-01-17

**Authors:** Katsuhiko Ono, Koki Nakagawa, Masaaki Tomura

**Affiliations:** a Department of Materials Science and Engineering, Nagoya Institute of Technology, Gokiso, Showa-ku, Nagoya 466-8555, Japan; b Institute for Molecular Science, Myodaiji, Okazaki 444-8585, Japan

## Abstract

The title compound, C_26_H_22_O_4_, is a derivative of 1,4-bis­(phenyl­ethyn­yl)benzene substituted by four meth­oxy groups on the terminal benzene rings. The mol­ecule is almost planar with an r.m.s. deviation of 0.266 Å. The dihedral angles between the two terminal benzene rings and the central benzene ring are 7.96 (6) and 13.32 (7)°. In the crystal structure, mol­ecules aggregate *via* C—H⋯O inter­actions, forming mol­ecular tapes along the *a* axis, which aggregate to form a herring-bone structure.

## Related literature

For the crystal structure of 1,4-bis­[(2,6-dimethoxy­phen­yl)ethyn­yl]benzene, see: Ono *et al.* (2008 ▶). For related sructures, including a 1,4-bis­(phenyl­ethyn­yl)benzene system, see: Watt *et al.* (2004 ▶); Li *et al.* (1998 ▶); Filatov & Petrukhina (2005 ▶).
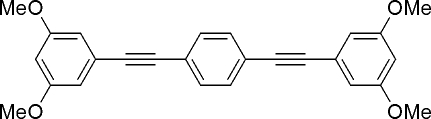


## Experimental

### 

#### Crystal data

C_26_H_22_O_4_
*M* *_r_* = 398.44
Monoclinic, 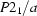

*a* = 8.8980 (5) Å
*b* = 19.4610 (8) Å
*c* = 12.2820 (5) Å
β = 100.607 (1)°
*V* = 2090.46 (17) Å^3^
*Z* = 4
Mo *K*α radiation
μ = 0.09 mm^−1^
*T* = 173 (1) K
0.30 × 0.25 × 0.15 mm

#### Data collection

Rigaku Mercury CCD diffractometer
Absorption correction: none
15238 measured reflections
4638 independent reflections
3914 reflections with *I* > 2σ(*I*)
*R*_int_ = 0.024

#### Refinement

*R*[*F* ^2^ > 2σ(*F* ^2^)] = 0.048
*wR*(*F* ^2^) = 0.140
*S* = 1.11
4638 reflections
271 parameters
H-atom parameters constrained
Δρ_max_ = 0.26 e Å^−3^
Δρ_min_ = −0.17 e Å^−3^

### 

Data collection: *CrystalClear* (Rigaku, 2001 ▶); cell refinement: *CrystalClear*; data reduction: *CrystalClear*; program(s) used to solve structure: *SIR2002* (Burla *et al.*, 2003 ▶); program(s) used to refine structure: *SHELXL97* (Sheldrick, 2008 ▶); molecular graphics: *PLATON* (Spek, 2003 ▶) and *Mercury* (Macrae *et al.*, 2006 ▶); software used to prepare material for publication: *WinGX* (Farrugia, 1999 ▶).

## Supplementary Material

Crystal structure: contains datablocks I, global. DOI: 10.1107/S160053680900155X/ci2753sup1.cif

Structure factors: contains datablocks I. DOI: 10.1107/S160053680900155X/ci2753Isup2.hkl

Additional supplementary materials:  crystallographic information; 3D view; checkCIF report

## Figures and Tables

**Table 1:** Hydrogen-bond geometry (Å, °)

*D*—H⋯*A*	*D*—H	H⋯*A*	*D*⋯*A*	*D*—H⋯*A*
C11—H11⋯O4^i^	0.95	2.42	3.3511 (17)	167
C14—H14⋯O2^ii^	0.95	2.37	3.2758 (16)	160

Symmetry codes: (i) 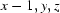; (ii) 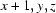.

**Fig. 1.**
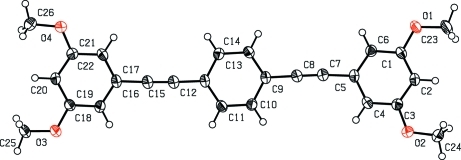
The molecular structure of (I), with atom labels and 50% probability displacement ellipsoids for non-H atoms and H atoms are shown as small spheres of arbitrary radii.

**Fig. 2.**
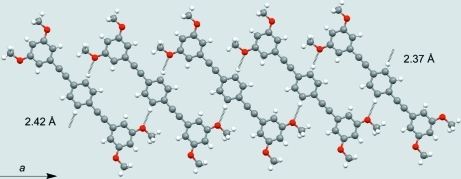
Partial packing diagram of (I), showing a molecular tape along the a axis.

**Fig. 3.**
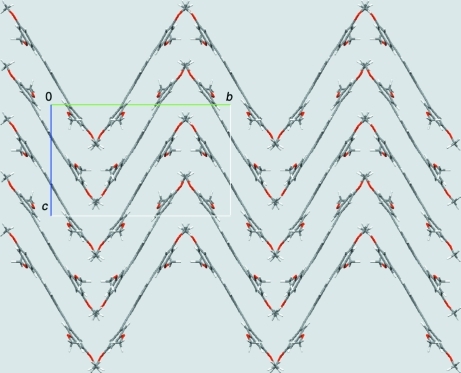
The packing diagram of (I), showing herringbone-type network on the bc plane.

## supplementary crystallographic information

### Comment

The synthetic research of ethynylated aromatic compounds has attracted considerable attention because of interests in their molecular structures, optical properties, and molecular electronics. Among these ethynylated aromatic compounds, 1,4-bis(phenylethynyl)benzene derivatives have been extensively studied. These compounds have stiff, linear molecular structures and are used as building blocks in the applications. Recently, we found that 1,4-bis[(2,6-dimethoxyphenyl)ethynyl]benzene, (II), formed a zigzag molecular network in the crystal (Ono *et al.*, 2008). The crystal structure is different from those of 1,4-bis(phenylethynyl)benzene derivatives (Watt *et al.*, 2004; Li *et al.*, 1998; Filatov & Petrukhina, 2005). With regard to this, we investigated the molecular and crystal structure of the title compound, (I), which is a regioisomer of (II). The substitution effect of four methoxy groups at the terminal benzene rings was studied.

The molecular structure of (I) is shown in Fig. 1. The molecule is almost planar with an r.m.s deviation of 0.266 Å. The dihedral angles between the terminal benzene rings and the central benzene ring are 7.96 (6)° (C1–C6) and 13.32 (7)° (C17–C22). The methoxy groups are coplanar with the attached benzene rings.

The crystal structure is characterized by a molecular tape along the *a* axis formed by C—H···O interactions (Table 1 and Fig. 2). The molecular tapes aggregate to form a herring-bone-type structure, as shown in Fig.3. The crystal structure of (I) is different from that of (II). The crystal structures of (I) and (II) indicate that the methoxy groups at terminal benzene rings play an important role in the crystal packing.

### Experimental

The title compound (I) was prepared as follows: Tetrakis(triphenylphosphine)palladium(0) [Pd(PPh_3_)_4_] (52 mg, 0.045 mmol) was added to a mixture of 1-ethynyl-3,5-dimethoxybenzene (0.39 g, 2.4 mmol), 1,4-diiodobenzene (0.39 g, 1.2 mmol) and copper(I) iodide (5 mg, 0.03 mmol) in dry triethylamine (7 ml) under nitrogen. The reaction mixture was stirred for 18 h at 353 K. After removal of the solvent, dichloromethane (20 ml) and aqueous disodium ethylenediaminetetraacetate (Na_2_edta) solution (5%, 20 ml) were added. The organic layer was separated and washed with water (20 ml). The organic solution was dried over Na_2_SO_4 _and concentrated. The residue was chromatographed on silica gel (CH_2_Cl_2_) to afford the title compound (0.23 g, 49%) as a yellow powder. Yellow crystals of the compound, suitable for X-ray analysis were grown from an ethanol solution.

### Refinement

All H atoms were placed in geometrically calculated positions, with C-H = 0.95 (aromatic) and 0.98 Å (methyl) and *U*_iso_(H) = 1.2*U*_eq_(C) (aromatic) and 1.5*U*_eq_(C) (methyl), and refined using a riding model.

### Crystal data

**Table:**

C_26_H_22_O_4_	*F*(000) = 840
*M**_r_* = 398.44	*D*_x_ = 1.266 Mg m^−^^3^
Monoclinic, *P*2_1_/*a*	Melting point: 431 K
Hall symbol: -P 2yab	Mo *K*α radiation, λ = 0.71073 Å
*a* = 8.8980 (5) Å	Cell parameters from 5437 reflections
*b* = 19.4610 (8) Å	θ = 3.1–27.5°
*c* = 12.2820 (5) Å	µ = 0.09 mm^−^^1^
β = 100.607 (1)°	*T* = 173 K
*V* = 2090.46 (17) Å^3^	Block, yellow
*Z* = 4	0.30 × 0.25 × 0.15 mm

### Data collection

**Table:**

Rigaku Mercury CCD diffractometer	3914 reflections with *I* > 2σ(*I*)
Radiation source: Rotating Anode	*R*_int_ = 0.024
Graphite Monochromator	θ_max_ = 27.5°, θ_min_ = 3.1°
Detector resolution: 14.7059 pixels mm^-1^	*h* = −11→10
φ and ω scans	*k* = −22→25
15238 measured reflections	*l* = −15→12
4638 independent reflections	

### Refinement

**Table:**

Refinement on *F*^2^	Primary atom site location: structure-invariant direct methods
Least-squares matrix: full	Secondary atom site location: difference Fourier map
*R*[*F*^2^ > 2σ(*F*^2^)] = 0.048	H-atom parameters constrained
*wR*(*F*^2^) = 0.140	*w* = 1/[σ^2^(*F*_o_^2^) + (0.0818*P*)^2^ + 0.1522*P*] where *P* = (*F*_o_^2^ + 2*F*_c_^2^)/3
*S* = 1.11	(Δ/σ)_max_ = 0.001
4638 reflections	Δρ_max_ = 0.26 e Å^−^^3^
271 parameters	Δρ_min_ = −0.17 e Å^−^^3^
0 restraints	

### Special details

**Table:**

Experimental. IR (KBr, cm^-1^): 1605, 1580, 1345, 1254, 1202, 1161, 1065, 841; ^1^H NMR (CDCl_3_, δ p.p.m.): 3.81 (s, 12H), 6.48 (t, J = 2.3 Hz, 2H), 6.70 (d, J = 2.3 Hz, 4H), 7.51 (s, 4H); ^13^C NMR (CDCl_3_, δ p.p.m.): 55.3, 88.6, 91.3, 102.0, 109.4, 123.0, 124.3, 131.6, 160.6; MS (EI): m/*z* 398 (*M*^+^), 199.
Geometry. All e.s.d.'s (except the e.s.d. in the dihedral angle between two l.s. planes) are estimated using the full covariance matrix. The cell e.s.d.'s are taken into account individually in the estimation of e.s.d.'s in distances, angles and torsion angles; correlations between e.s.d.'s in cell parameters are only used when they are defined by crystal symmetry. An approximate (isotropic) treatment of cell e.s.d.'s is used for estimating e.s.d.'s involving l.s. planes.
Refinement. Refinement of *F*^2^ against ALL reflections. The weighted *R*-factor *wR* and goodness of fit *S* are based on *F*^2^, conventional *R*-factors *R* are based on *F*, with *F* set to zero for negative *F*^2^. The threshold expression of *F*^2^ > σ(*F*^2^) is used only for calculating *R*-factors(gt) *etc*. and is not relevant to the choice of reflections for refinement. *R*-factors based on *F*^2^ are statistically about twice as large as those based on *F*, and *R*- factors based on ALL data will be even larger.

### Fractional atomic coordinates and isotropic or equivalent isotropic displacement parameters (Å^2^)

**Table:**

	*x*	*y*	*z*	*U*_iso_*/*U*_eq_	
C1	0.04347 (14)	0.17522 (6)	0.71287 (10)	0.0266 (3)	
C2	−0.11459 (14)	0.17481 (6)	0.67760 (11)	0.0266 (3)	
H2	−0.1799	0.1980	0.7185	0.032*	
C3	−0.17430 (13)	0.13932 (6)	0.58033 (11)	0.0265 (3)	
C4	−0.07979 (14)	0.10623 (6)	0.51948 (11)	0.0277 (3)	
H4	−0.1225	0.0824	0.4535	0.033*	
C5	0.07839 (14)	0.10787 (6)	0.55512 (10)	0.0261 (3)	
C6	0.14059 (14)	0.14194 (6)	0.65309 (11)	0.0283 (3)	
H6	0.2480	0.1424	0.6787	0.034*	
C7	0.17340 (14)	0.07642 (6)	0.48619 (11)	0.0288 (3)	
C8	0.24292 (14)	0.05168 (7)	0.42138 (11)	0.0288 (3)	
C9	0.32492 (14)	0.02180 (6)	0.34289 (10)	0.0262 (3)	
C10	0.24597 (14)	−0.01164 (7)	0.24864 (11)	0.0286 (3)	
H10	0.1377	−0.0150	0.2371	0.034*	
C11	0.32439 (14)	−0.03986 (7)	0.17214 (11)	0.0303 (3)	
H11	0.2695	−0.0621	0.1081	0.036*	
C12	0.48372 (14)	−0.03594 (6)	0.18839 (10)	0.0273 (3)	
C13	0.56275 (14)	−0.00329 (6)	0.28348 (11)	0.0297 (3)	
H13	0.6712	−0.0009	0.2959	0.036*	
C14	0.48439 (14)	0.02550 (7)	0.35943 (11)	0.0295 (3)	
H14	0.5392	0.0479	0.4233	0.035*	
C15	0.56748 (14)	−0.06453 (7)	0.10994 (11)	0.0305 (3)	
C16	0.64138 (15)	−0.08764 (7)	0.04662 (11)	0.0313 (3)	
C17	0.73414 (14)	−0.11338 (6)	−0.02860 (11)	0.0289 (3)	
C18	0.67256 (14)	−0.15780 (7)	−0.11433 (11)	0.0309 (3)	
H18	0.5689	−0.1720	−0.1231	0.037*	
C19	0.76419 (14)	−0.18118 (7)	−0.18695 (11)	0.0289 (3)	
C20	0.91672 (14)	−0.16179 (7)	−0.17484 (11)	0.0289 (3)	
H20	0.9792	−0.1787	−0.2238	0.035*	
C21	0.97568 (14)	−0.11696 (7)	−0.08931 (11)	0.0310 (3)	
C22	0.88622 (14)	−0.09254 (7)	−0.01627 (11)	0.0316 (3)	
H22	0.9283	−0.0619	0.0416	0.038*	
C23	0.02302 (18)	0.24526 (8)	0.87014 (12)	0.0434 (4)	
H23A	0.0889	0.2677	0.9328	0.065*	
H23B	−0.0440	0.2122	0.8980	0.065*	
H23C	−0.0392	0.2800	0.8247	0.065*	
C24	−0.43253 (15)	0.17035 (8)	0.58918 (13)	0.0409 (4)	
H24A	−0.5365	0.1618	0.5488	0.061*	
H24B	−0.4108	0.2197	0.5890	0.061*	
H24C	−0.4236	0.1543	0.6657	0.061*	
C25	0.77935 (17)	−0.24753 (8)	−0.34851 (12)	0.0398 (3)	
H25A	0.7151	−0.2772	−0.4025	0.060*	
H25B	0.8679	−0.2737	−0.3107	0.060*	
H25C	0.8146	−0.2082	−0.3868	0.060*	
C26	1.22202 (17)	−0.11600 (9)	−0.14105 (15)	0.0506 (4)	
H26A	1.3229	−0.0950	−0.1178	0.076*	
H26B	1.1803	−0.1025	−0.2175	0.076*	
H26C	1.2316	−0.1661	−0.1366	0.076*	
O1	0.11541 (10)	0.21008 (5)	0.80454 (8)	0.0360 (2)	
O2	−0.32622 (10)	0.13442 (5)	0.53688 (8)	0.0352 (2)	
O3	0.69301 (10)	−0.22363 (5)	−0.26936 (9)	0.0396 (3)	
O4	1.12292 (11)	−0.09341 (6)	−0.07075 (9)	0.0477 (3)	

### Atomic displacement parameters (Å^2^)

**Table:**

	*U*^11^	*U*^22^	*U*^33^	*U*^12^	*U*^13^	*U*^23^
C1	0.0340 (6)	0.0247 (6)	0.0213 (6)	0.0006 (5)	0.0059 (5)	−0.0006 (5)
C2	0.0318 (6)	0.0241 (6)	0.0263 (7)	0.0011 (5)	0.0113 (5)	−0.0004 (5)
C3	0.0273 (6)	0.0244 (6)	0.0287 (7)	−0.0013 (4)	0.0073 (5)	0.0015 (5)
C4	0.0345 (6)	0.0257 (6)	0.0235 (6)	−0.0007 (5)	0.0068 (5)	−0.0036 (5)
C5	0.0322 (6)	0.0232 (6)	0.0249 (6)	0.0034 (5)	0.0106 (5)	0.0016 (5)
C6	0.0280 (6)	0.0281 (6)	0.0291 (7)	0.0025 (5)	0.0058 (5)	−0.0005 (5)
C7	0.0314 (6)	0.0279 (6)	0.0277 (7)	0.0016 (5)	0.0071 (5)	−0.0007 (5)
C8	0.0324 (6)	0.0280 (6)	0.0266 (7)	0.0020 (5)	0.0069 (5)	−0.0004 (5)
C9	0.0309 (6)	0.0248 (6)	0.0243 (6)	0.0033 (5)	0.0089 (5)	0.0012 (5)
C10	0.0266 (6)	0.0331 (7)	0.0265 (7)	0.0027 (5)	0.0061 (5)	−0.0005 (5)
C11	0.0340 (6)	0.0338 (7)	0.0226 (6)	0.0014 (5)	0.0042 (5)	−0.0045 (5)
C12	0.0330 (6)	0.0269 (6)	0.0238 (6)	0.0043 (5)	0.0103 (5)	0.0000 (5)
C13	0.0277 (6)	0.0333 (7)	0.0293 (7)	0.0007 (5)	0.0083 (5)	−0.0012 (5)
C14	0.0325 (6)	0.0316 (6)	0.0248 (7)	−0.0015 (5)	0.0064 (5)	−0.0051 (5)
C15	0.0346 (6)	0.0303 (7)	0.0278 (7)	0.0033 (5)	0.0086 (5)	−0.0006 (5)
C16	0.0358 (6)	0.0316 (7)	0.0278 (7)	0.0036 (5)	0.0092 (5)	−0.0007 (5)
C17	0.0344 (6)	0.0299 (6)	0.0238 (6)	0.0062 (5)	0.0089 (5)	0.0002 (5)
C18	0.0286 (6)	0.0338 (7)	0.0322 (7)	−0.0007 (5)	0.0109 (5)	−0.0034 (6)
C19	0.0317 (6)	0.0294 (6)	0.0259 (7)	−0.0011 (5)	0.0065 (5)	−0.0044 (5)
C20	0.0296 (6)	0.0330 (7)	0.0255 (7)	0.0018 (5)	0.0091 (5)	−0.0038 (5)
C21	0.0286 (6)	0.0363 (7)	0.0285 (7)	−0.0008 (5)	0.0057 (5)	−0.0029 (5)
C22	0.0356 (7)	0.0348 (7)	0.0244 (7)	0.0003 (5)	0.0057 (5)	−0.0071 (5)
C23	0.0512 (8)	0.0486 (9)	0.0291 (8)	0.0112 (7)	0.0037 (6)	−0.0135 (7)
C24	0.0286 (6)	0.0471 (9)	0.0487 (9)	0.0001 (6)	0.0112 (6)	−0.0110 (7)
C25	0.0459 (8)	0.0429 (8)	0.0324 (8)	−0.0052 (6)	0.0121 (6)	−0.0150 (6)
C26	0.0339 (7)	0.0718 (12)	0.0499 (10)	−0.0131 (7)	0.0179 (7)	−0.0190 (8)
O1	0.0364 (5)	0.0429 (6)	0.0274 (5)	0.0056 (4)	0.0023 (4)	−0.0123 (4)
O2	0.0271 (4)	0.0380 (5)	0.0403 (6)	−0.0022 (4)	0.0062 (4)	−0.0102 (4)
O3	0.0361 (5)	0.0459 (6)	0.0390 (6)	−0.0091 (4)	0.0123 (4)	−0.0198 (5)
O4	0.0302 (5)	0.0698 (7)	0.0446 (7)	−0.0122 (5)	0.0112 (4)	−0.0260 (6)

### Geometric parameters (Å, °)

**Table:**

C1—O1	1.3684 (15)	C17—C22	1.3940 (18)
C1—C6	1.3924 (16)	C17—C18	1.3942 (18)
C1—C2	1.3933 (17)	C18—C19	1.3911 (16)
C2—C3	1.3975 (18)	C18—H18	0.95
C2—H2	0.95	C19—O3	1.3683 (15)
C3—O2	1.3622 (15)	C19—C20	1.3900 (17)
C3—C4	1.3826 (16)	C20—C21	1.3917 (18)
C4—C5	1.3953 (17)	C20—H20	0.95
C4—H4	0.95	C21—O4	1.3670 (15)
C5—C6	1.3960 (18)	C21—C22	1.3882 (17)
C5—C7	1.4386 (16)	C22—H22	0.95
C6—H6	0.95	C23—O1	1.4274 (16)
C7—C8	1.1956 (17)	C23—H23A	0.98
C8—C9	1.4348 (16)	C23—H23B	0.98
C9—C14	1.3977 (17)	C23—H23C	0.98
C9—C10	1.3991 (18)	C24—O2	1.4213 (15)
C10—C11	1.3831 (17)	C24—H24A	0.98
C10—H10	0.95	C24—H24B	0.98
C11—C12	1.3969 (17)	C24—H24C	0.98
C11—H11	0.95	C25—O3	1.4236 (16)
C12—C13	1.3999 (18)	C25—H25A	0.98
C12—C15	1.4345 (16)	C25—H25B	0.98
C13—C14	1.3820 (17)	C25—H25C	0.98
C13—H13	0.95	C26—O4	1.4130 (17)
C14—H14	0.95	C26—H26A	0.98
C15—C16	1.1949 (18)	C26—H26B	0.98
C16—C17	1.4375 (17)	C26—H26C	0.98
			
O1—C1—C6	115.01 (10)	C19—C18—C17	119.46 (11)
O1—C1—C2	123.44 (11)	C19—C18—H18	120.3
C6—C1—C2	121.49 (11)	C17—C18—H18	120.3
C1—C2—C3	118.14 (11)	O3—C19—C20	123.57 (11)
C1—C2—H2	120.9	O3—C19—C18	115.19 (11)
C3—C2—H2	120.9	C20—C19—C18	121.24 (12)
O2—C3—C4	114.46 (11)	C19—C20—C21	118.42 (11)
O2—C3—C2	124.28 (11)	C19—C20—H20	120.8
C4—C3—C2	121.26 (11)	C21—C20—H20	120.8
C3—C4—C5	119.91 (11)	O4—C21—C22	115.05 (12)
C3—C4—H4	120.0	O4—C21—C20	123.55 (11)
C5—C4—H4	120.0	C22—C21—C20	121.40 (11)
C4—C5—C6	119.88 (11)	C21—C22—C17	119.40 (12)
C4—C5—C7	118.29 (11)	C21—C22—H22	120.3
C6—C5—C7	121.78 (11)	C17—C22—H22	120.3
C1—C6—C5	119.30 (11)	O1—C23—H23A	109.5
C1—C6—H6	120.4	O1—C23—H23B	109.5
C5—C6—H6	120.4	H23A—C23—H23B	109.5
C8—C7—C5	174.44 (14)	O1—C23—H23C	109.5
C7—C8—C9	179.42 (15)	H23A—C23—H23C	109.5
C14—C9—C10	119.07 (11)	H23B—C23—H23C	109.5
C14—C9—C8	120.64 (11)	O2—C24—H24A	109.5
C10—C9—C8	120.29 (11)	O2—C24—H24B	109.5
C11—C10—C9	120.50 (11)	H24A—C24—H24B	109.5
C11—C10—H10	119.8	O2—C24—H24C	109.5
C9—C10—H10	119.8	H24A—C24—H24C	109.5
C10—C11—C12	120.45 (11)	H24B—C24—H24C	109.5
C10—C11—H11	119.8	O3—C25—H25A	109.5
C12—C11—H11	119.8	O3—C25—H25B	109.5
C11—C12—C13	119.00 (11)	H25A—C25—H25B	109.5
C11—C12—C15	121.46 (11)	O3—C25—H25C	109.5
C13—C12—C15	119.54 (11)	H25A—C25—H25C	109.5
C14—C13—C12	120.58 (11)	H25B—C25—H25C	109.5
C14—C13—H13	119.7	O4—C26—H26A	109.5
C12—C13—H13	119.7	O4—C26—H26B	109.5
C13—C14—C9	120.39 (12)	H26A—C26—H26B	109.5
C13—C14—H14	119.8	O4—C26—H26C	109.5
C9—C14—H14	119.8	H26A—C26—H26C	109.5
C16—C15—C12	177.95 (14)	H26B—C26—H26C	109.5
C15—C16—C17	177.90 (14)	C1—O1—C23	118.13 (10)
C22—C17—C18	120.08 (11)	C3—O2—C24	118.91 (10)
C22—C17—C16	119.31 (12)	C19—O3—C25	117.90 (10)
C18—C17—C16	120.60 (11)	C21—O4—C26	118.72 (11)
			
O1—C1—C2—C3	177.69 (11)	C8—C9—C14—C13	−179.92 (12)
C6—C1—C2—C3	0.59 (18)	C22—C17—C18—C19	0.2 (2)
C1—C2—C3—O2	179.60 (11)	C16—C17—C18—C19	179.07 (12)
C1—C2—C3—C4	−0.90 (18)	C17—C18—C19—O3	−178.99 (12)
O2—C3—C4—C5	179.63 (11)	C17—C18—C19—C20	0.8 (2)
C2—C3—C4—C5	0.08 (18)	O3—C19—C20—C21	178.44 (12)
C3—C4—C5—C6	1.06 (18)	C18—C19—C20—C21	−1.4 (2)
C3—C4—C5—C7	−176.30 (11)	C19—C20—C21—O4	−178.72 (13)
O1—C1—C6—C5	−176.80 (11)	C19—C20—C21—C22	0.9 (2)
C2—C1—C6—C5	0.53 (19)	O4—C21—C22—C17	179.75 (12)
C4—C5—C6—C1	−1.36 (18)	C20—C21—C22—C17	0.1 (2)
C7—C5—C6—C1	175.90 (11)	C18—C17—C22—C21	−0.6 (2)
C14—C9—C10—C11	−0.84 (19)	C16—C17—C22—C21	−179.55 (12)
C8—C9—C10—C11	179.30 (12)	C6—C1—O1—C23	−179.78 (12)
C9—C10—C11—C12	0.59 (19)	C2—C1—O1—C23	2.94 (18)
C10—C11—C12—C13	0.28 (19)	C4—C3—O2—C24	−175.99 (12)
C10—C11—C12—C15	−179.69 (12)	C2—C3—O2—C24	3.55 (18)
C11—C12—C13—C14	−0.90 (19)	C20—C19—O3—C25	−1.8 (2)
C15—C12—C13—C14	179.07 (12)	C18—C19—O3—C25	178.03 (12)
C12—C13—C14—C9	0.65 (19)	C22—C21—O4—C26	179.71 (14)
C10—C9—C14—C13	0.22 (19)	C20—C21—O4—C26	−0.6 (2)

### Hydrogen-bond geometry (Å, °)

**Table:**

*D*—H···*A*	*D*—H	H···*A*	*D*···*A*	*D*—H···*A*
C11—H11···O4^i^	0.95	2.42	3.3511 (17)	167
C14—H14···O2^ii^	0.95	2.37	3.2758 (16)	160

Symmetry codes: (i) *x*−1, *y*, *z*; (ii) *x*+1, *y*, *z*.

## References

[bb1] Burla, M. C., Camalli, M., Carrozzini, B., Cascarano, G. L., Giacovazzo, C., Polidori, G. & Spagna, R. (2003). *J. Appl. Cryst.* **36**, 1103.

[bb2] Farrugia, L. J. (1999). *J. Appl. Cryst.* **32**, 837–838.

[bb3] Filatov, A. S. & Petrukhina, M. A. (2005). *Acta Cryst.* C**61**, o193–o194. 10.1107/S0108270105000983 15750253

[bb4] Li, H., Powell, D. R., Firman, T. K. & West, R. (1998). *Macromolecules*, **31**, 1093–1098.

[bb5] Macrae, C. F., Edgington, P. R., McCabe, P., Pidcock, E., Shields, G. P., Taylor, R., Towler, M. & van de Streek, J. (2006). *J. Appl. Cryst.* **39**, 453–457.

[bb6] Ono, K., Tsukamoto, K., Tomura, M. & Saito, K. (2008). *Acta Cryst.* E**64**, o1069. 10.1107/S1600536808013664 PMC2961624 21202588

[bb7] Rigaku (2001). *CrystalClear* Rigaku Corporation, Tokyo, Japan.

[bb8] Sheldrick, G. M. (2008). *Acta Cryst.* A**64**, 112–122. 10.1107/S0108767307043930 18156677

[bb9] Spek, A. L. (2003). *J. Appl. Cryst.* **36**, 7–13.

[bb10] Watt, S. W., Dai, C., Scott, A. J., Burke, J. M., Thomas, R. Ll., Collings, J. C., Viney, C., Clegg, W. & Marder, T. B. (2004). *Angew. Chem. Int. Ed.* **43**, 3061–3063. 10.1002/anie.200453828 15188481

